# Enhancement of arterial pulsation during flow-mediated dilation is impaired in the presence of ischemic heart disease

**DOI:** 10.1186/s40064-016-2794-0

**Published:** 2016-07-16

**Authors:** Eisuke Amiya, Masafumi Watanabe, Shogo Watanabe, Munenori Takata, Issei Komuro

**Affiliations:** Department of Cardiovascular Medicine, Graduate School of Medicine, The University of Tokyo, 7-3-1 Hongo, Bunkyo-ku, Tokyo 113-8655 Japan; Department of Clinical Research Support Center, The University of Tokyo Hospital, Tokyo, Japan; Department of Pathophysiological Laboratory Sciences, Nagoya University Graduate School of Medicine, Nagoya, Aichi Japan

**Keywords:** Arterial pulsation, Flow-mediated dilation, Ischemic heart disease, Vascular ultrasound

## Abstract

**Purpose:**

The aim of this study is to investigate the relationship between arterial pulse amplitude change under increased shear stress and the presence of ischemic heart disease (IHD).

**Methods:**

This study comprised 31 subjects, including 14 subjects with IHD. We investigated the change in brachial artery pulse amplitude during flow-mediated dilation (FMD) using ultrasonography.

**Results:**

The arterial pulse amplitude increased during FMD in 19 subjects, whereas it decreased in 12 subjects. There was a marked difference in the change in arterial pulse amplitude (the maximum amplitude of the arterial pulse amplitude during FMD/the arterial pulse amplitude at baseline) between subjects with and without IHD (0.98 ± 0.53 and 1.37 ± 0.53, *p* = 0.028). Furthermore, decreased arterial pulse amplitude during FMD was a significant predictor of IHD after adjustment of age, blood pressure, the presence of each type of coronary risks, the value of FMD and sex (*p* = 0.0001).

**Conclusions:**

The decrease of arterial pulsation amplitude during FMD was a useful predictive parameter for IHD.

**Electronic supplementary material:**

The online version of this article (doi:10.1186/s40064-016-2794-0) contains supplementary material, which is available to authorized users.

## Background

Shear stress is one of the potent factors related with the progression of atherosclerosis (Samady et al. 2011). Low shear stress leads to a proatherogenic vascular condition, which subsequently leads to the focal development of atherosclerosis in early lesions (Koskinas et al. 2009). In contrast, high shear stress has been shown to enhance plaque vulnerability in the later stage of atherosclerosis (Kenagy et al. 2002). If there is a stenotic lesion in a coronary artery that increases the shear stress at the stenotic site (Thim et al. 2012; Vita et al. 1989), the vasculature responds to this flow-limiting condition and compensates for it by arterial remodeling, resulting in a decrease in shear stress (Vita et al. 1989; Silver and Vita 2006). On the other hand, this compensation response is lost in dysfunctional vasculature, resulting in failure to control shear stress and the progression of atherosclerosis. Therefore, the ability to compensate for altered shear stress is considered to correspond to the risk of atherosclerosis progression.

Multiple aspects of vascular function have been reported to relate to atherosclerotic processes (Bonetti et al. 2003). Among them, flow-mediated dilation (FMD) is one of the methods used to evaluate the state of endothelial cell function (Watanabe et al. 2013). During FMD measurements, increased blood flow after vascular occlusion is required to stimulate endothelial cells. This situation shares similarity with atherosclerotic vessel stenosis, with respect to the increase in shear stress. Thus, vascular behavior during FMD is presumed to correlate with the compensative vascular response to the presence of high shear stress lesions. Therefore, the documentation of vascular behavior in the presence of increased flow and shear stress during FMD is considered to be useful information to predict the process of atherosclerosis. FMD is a baseline diameter behavior under high shear stress; however, it only reflects one specific hemodynamic movement in a limited manner, and vascular functions observed by other modalities may complement its predictive power for atherosclerotic diseases (Watanabe et al. 2014).

Arterial pulsation is produced by an increase in blood pressure during systole. The amplitude of the pulsatile dilation is determined by the arterial wall stiffness, its vascular tone, and pulse pressure (Budoff et al. 2003; Giannattasio and Mancia 2002). Arterial pulsation behavior can be observed using echographic serial monitoring or magnetic resonance modalities (Voges et al. 2012); however, its characteristics have not been well elucidated.

Recently, it has been shown that arterial stiffness, which is measured by pulse wave velocity (PWV), can change during the FMD process (Naka et al. 2006). Flow-mediated changes in the PWV have been reported to be impaired in subjects with endothelial dysfunction, when compared with normal controls. In this manner, impairment of the ability to change vascular behavior during FMD seems to correspond to the presence of cardiovascular disease. However, the relationship between vascular disease and the behavior of arterial pulsation during FMD has not been well documented. The objective of this study was to compare the behavior of arterial pulsation in subjects with and without ischemic heart disease (IHD) and clarify the association of vascular behaviors during arterial pulsation in the presence of ischemic heart disease.

## Methods

### Subjects

This study enrolled 31 subjects who were hospitalized in the Cardiovascular Department at the University of Tokyo Hospital. The study subjects were recruited from the consecutive patients of hospitalization for the diagnostic cardiac catheterization for IHD and diagnostic electrophysiological study for arrhythmia, whose informed consent was obtained. The subjects whose results of arterial pulse tracing were poor image and cannot be analyzed were excluded (we excluded 12 subjects). As a result, the study subjects included 17 subjects without IHD and 14 subjects with IHD. IHD was defined as the presence of at least one of the following: >50 % luminal diameter narrowing of more than one epicardial coronary artery on angiography, a history of coronary revascularization, or a history of myocardial infarction. The exclusion criterion was the presence of unstable clinical conditions. All components of standard informed consent, including the purpose of the study, the risk, and the benefits, were fully explained. Written informed consent was obtained from each subject. The study protocol conformed to the tenets of the Declaration of Helsinki and was reviewed and approved by the University of Tokyo Institutional Review Board (3003).

### FMD measurement

FMD was measured in accordance with the method established in a publication by the International Brachial Artery Reactivity Task Force (Corretti et al. 2002). The subjects were instructed to abstain from eating, smoking, and caffeine consumption for at least 4 h prior to the start of the study and to lie down for 20 min. FMD of the brachial artery was measured using amplitude- and brightness-mode ultrasonography with a linear array 10-MHz transducer (UNEXEF18G; UNEX, Nagoya, Japan). After collecting baseline diameter measurements for 30 s, the cuff was inflated to 50 mmHg above the patient’s systolic blood pressure, maintained at this pressure for 5 min, and then deflated (Watanabe et al. 2013). The diameter of the brachial artery was continuously recorded for 2 min after the cuff was deflated. The diameter was always measured during the end-diastolic phase, defined as the beginning of the R wave in the electrocardiogram. FMD was calculated as the percentage change in artery diameter from the baseline value before cuff release to the peak value after cuff release.

### Arterial pulse amplitude

**Fig. 1:**
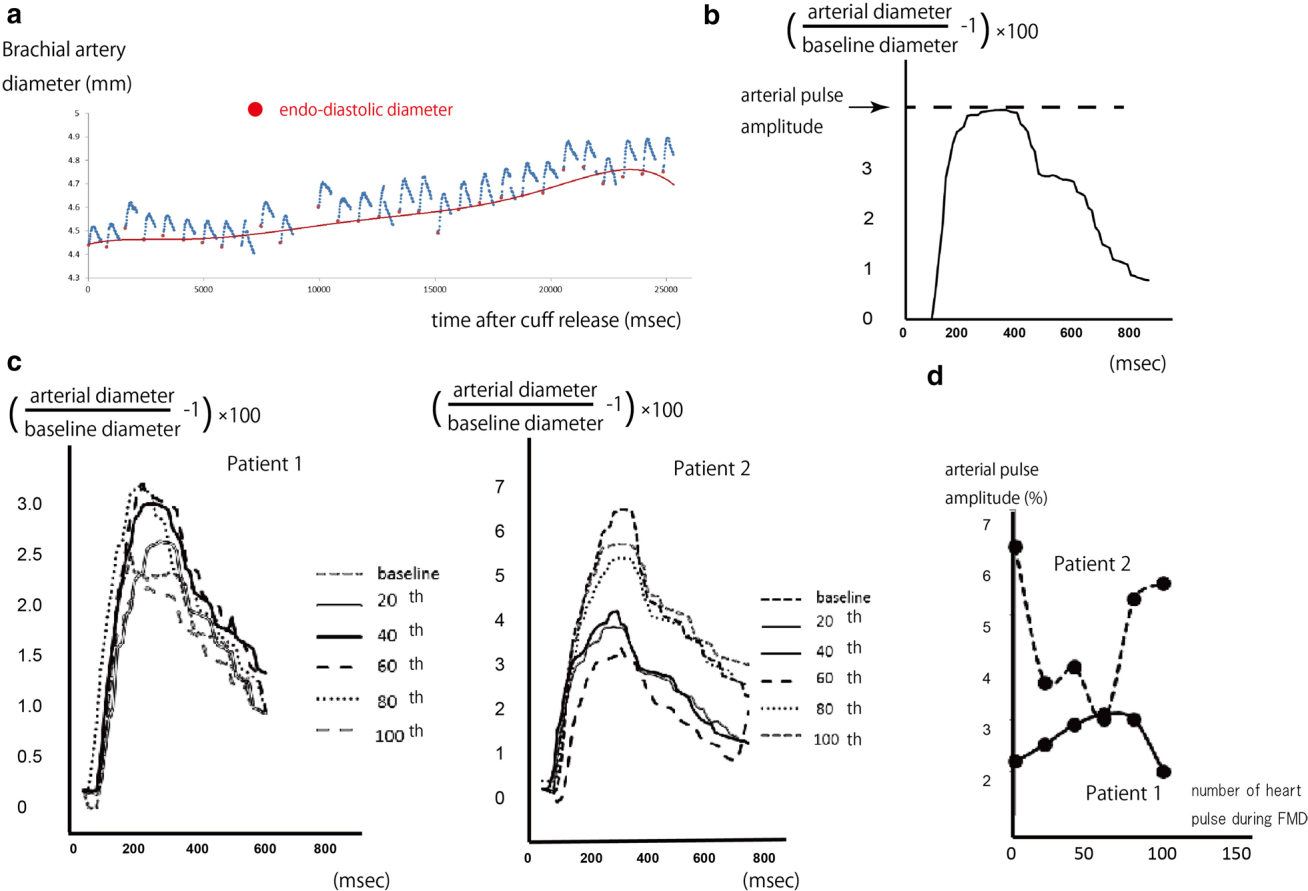
**a** Continuous monitoring of the arterial pulsation waveform during flow-mediated dilation (FMD). The arterial diameter was monitored using amplitude- and brightness-mode ultrasonography with a linear array 10-MHz transducer (UNEXEF18G, UNEX, Nagoya, Japan). **b** Definition of the arterial pulse amplitude. The change in arterial diameter over the course of one cardiac cycle is presented. **c** Two characteristic examples of arterial pulsation behavior during FMD. The arterial pulse amplitude increased during FMD in Patient 1, whereas it decreased in Patient 2. **d** The arterial pulse amplitude is plotted along the time course of FMD for Patients 1 and 2

We investigated the change in brachial artery pulsation during hyperemia-induced vasodilation using amplitude- and brightness-mode ultrasonography with a linear array 10-MHz transducer (UNEXEF18G; UNEX, Nagoya, Japan). Each arterial pulsation waveform was measured and standardized to the value of the end-diastolic diameter of each pulsation. Figure 1a shows an example waveform from one of the subjects. The arterial pulse amplitude (%) was defined as: 100 × ([the maximum diameter of the brachial artery during one cardiac cycle]/[the end-diastolic diameter during the same cardiac cycle] − 1) (Fig. 1b). The intraclass correlation coefficient of the arterial pulse amplitude was 0.882 (N = 16). The waveforms at rest and at the 20th, 40th, 60th, 80th, 100th, and 120th heart pulses were compared after deflation of the cuff during FMD measurement (Fig. 1c). Two examples of this waveform series during FMD are presented in Fig. 1c. These examples show that the arterial pulse amplitude was enhanced during hyperemia-induced shear stress stimulation in the dilating phase of the end-diastolic diameter in Patient 1, whereas the arterial pulse amplitude decreased in Patient 2. The peak amplitude of arterial pulsation was traced during the process of FMD (Fig. 1d). The end-diastolic diameter gradually dilated during FMD, but the range of dilation was too small; thus, it did not explain the enhancement in arterial pulsation. The arterial diameter was recorded every 13 ms and it was traced 650 ms after the Q wave in each pulse.

### Statistics

Data are presented as the mean ± standard deviation. Differences between groups were evaluated using the Mann–Whitney *U* test, *t* test, and *χ*^2^ test. Potential relationships between parameters were explored using Pearson’s correlation test. A logistic regression analysis was performed to determine whether or not the decrease in arterial pulse amplitude during FMD remained a predictor of IHD even after adjustment for age, FMD, the value of arterial pulsation, coronary risk factors, and sex. The results of comparison are represented as box plots (middle hash of the box indicating the median; 25th–75th percentiles represented by end caps of the box; whiskers extend to the last observed value). A *p* value <0.05 was considered to be statistically significant. Data analyses were performed using the PASW Statistics 18 (SPSS Inc., Chicago, IL, USA) and JMP Pro 9 (SAS Institute, Cary, NC, USA) software packages.

## Results

The background characteristics of the subjects with and without IHD are presented in Table 1. There were no significant differences in blood pressure, heart rate, or several clinical parameters among the subjects with and without IHD, whereas the ratio of hypertension and the prescription of calcium blockers (41 vs 90 %) and beta blockers (29 vs 78 %) were significantly higher in subjects with IHD. In terms of vascular function parameters, the values of FMD and arterial pulse amplitude were not significantly different between subjects with and without IHD. Indeed, none of the coronary risk factors (diabetes, hypertension, advanced age, or hyperlipidemia), medications (beta blockers or calcium blockers), or other clinical parameters had any association with the value of arterial pulse amplitude (not shown). Age is an only parameter which associated with arterial pulsation amplitude (*R* = 0.38, *p* = 0.036)

**Table 1:** Patient characteristics in subjects with and without IHD

	IHD (−)	IHD (+)	
N (male/female)	17 (5/12)	14 (13/1)	
Age (years)	62.1 ± 15.1	69.5 ± 7.8	NS
Systolic BP (mmHg)	129.3 ± 25.4	130.0 ± 16.2	NS
Diastolic BP (mmHg)	67.4 ± 12.7	72.8 ± 11.6	NS
Pulse pressure (mmHg)	62.2 ± 22.2	57.2 ± 12.0	NS
Heart rate (/min)	69.3 ± 14.6	63.9 ± 11.1	NS
Diabetes	3 (17 %)	5 (35 %)	NS
Hypertension	6 (35.3 %)	14 (100 %)	*p* = 0.0005
Dyslipidemia	9 (52 %)	11 (78 %)	NS
Beta-blocker	5 (29 %)	11 (78 %)	*p* = 0.0052
Calcium blocker	7 (41 %)	13 (93 %)	*p* = 0.0015
Arterial pulse amplitude	2.9 ± 1.4	3.5 ± 1.8	NS
FMD (%)	6.2 ± 3.7	4.2 ± 2.5	NS
Brachial artery diameter (mm)	3.9 ± 0.7	4.1 ± 0.6	NS

*IHD* ischemic heart disease, *FMD* flow-mediated dilation, *BP* blood pressure, *NS* not significant

**Fig. 2:**
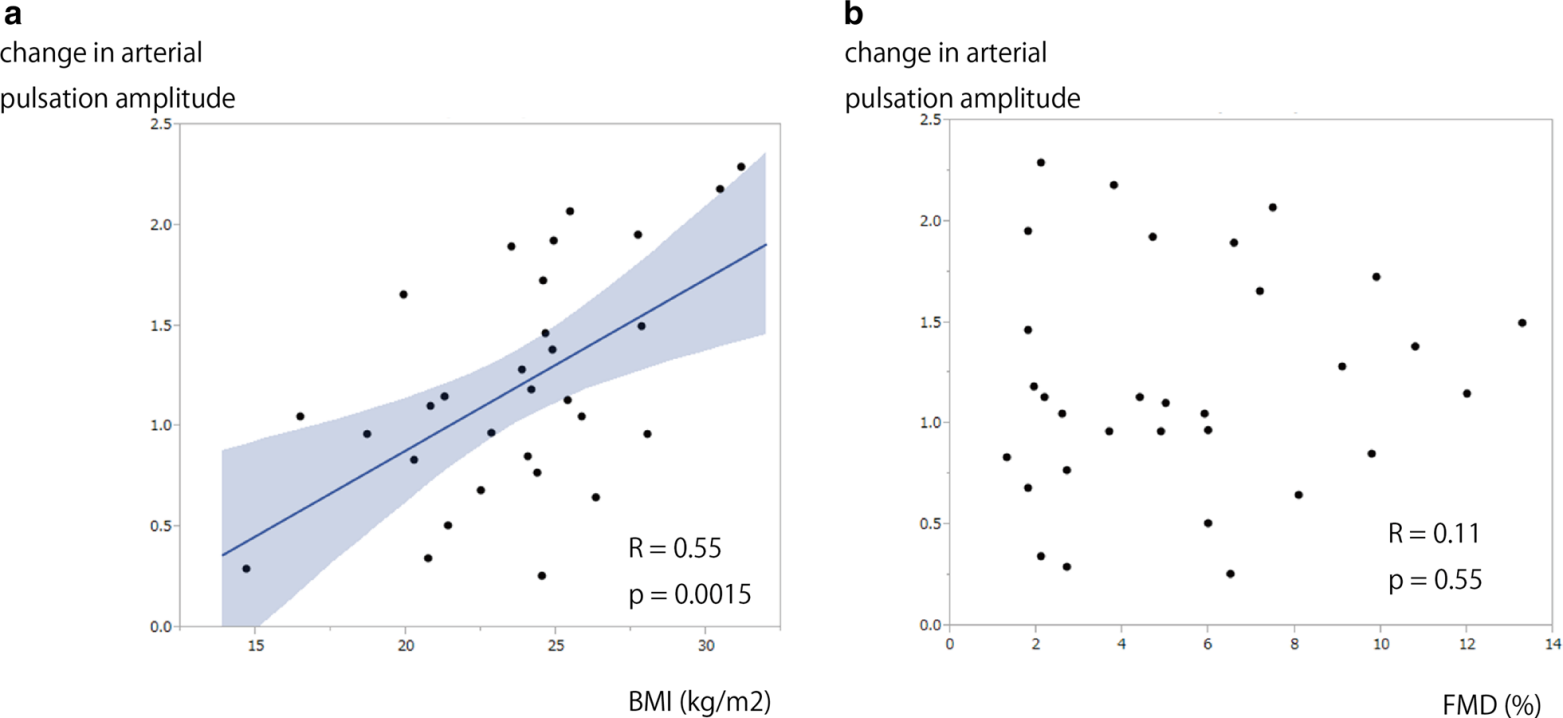
*Scatter plot* of the change in arterial pulse amplitude and body mass index (BMI) (**a**) and flow-mediated dilation (FMD) (**b**)

Next we investigated the change in arterial pulse amplitude during FMD (Fig. 1c, d). The change in this amplitude usually increased during the first time course of FMD and returned to baseline about 90 s later (as for Patient 1 in Fig. 1c). We defined the change in arterial pulse amplitude as the maximum change of the arterial pulse amplitude during FMD divided by the arterial pulse amplitude at baseline. The arterial pulse amplitude during FMD increased in 19 subjects and decreased in 12 subjects. The change in the arterial pulse amplitude was strongly associated with the arterial pulse amplitude at baseline (*R* = −0.73, *p* < 0.0001), brachial artery diameter (*R* = 0.37, *p* = 0.038) and body mass index (BMI) (*R* = 0.55, *p* = 0.015) (Fig. 2a). However, the other clinical parameters, including FMD (Fig. 2b), were not significantly correlated with the change in arterial pulse amplitude. In addition, the presence of coronary risk factors and the prescription of calcium blockers (1.26 ± 0.63 (off) vs 1.18 ± 0.55 (on), *p* = 0.66) or beta blockers (1.35 ± 0.49 (off) vs 1.08 ± 0.62 (on), *p* = 0.15) did not affect the change in arterial pulsation amplitude.

**Fig. 3:**
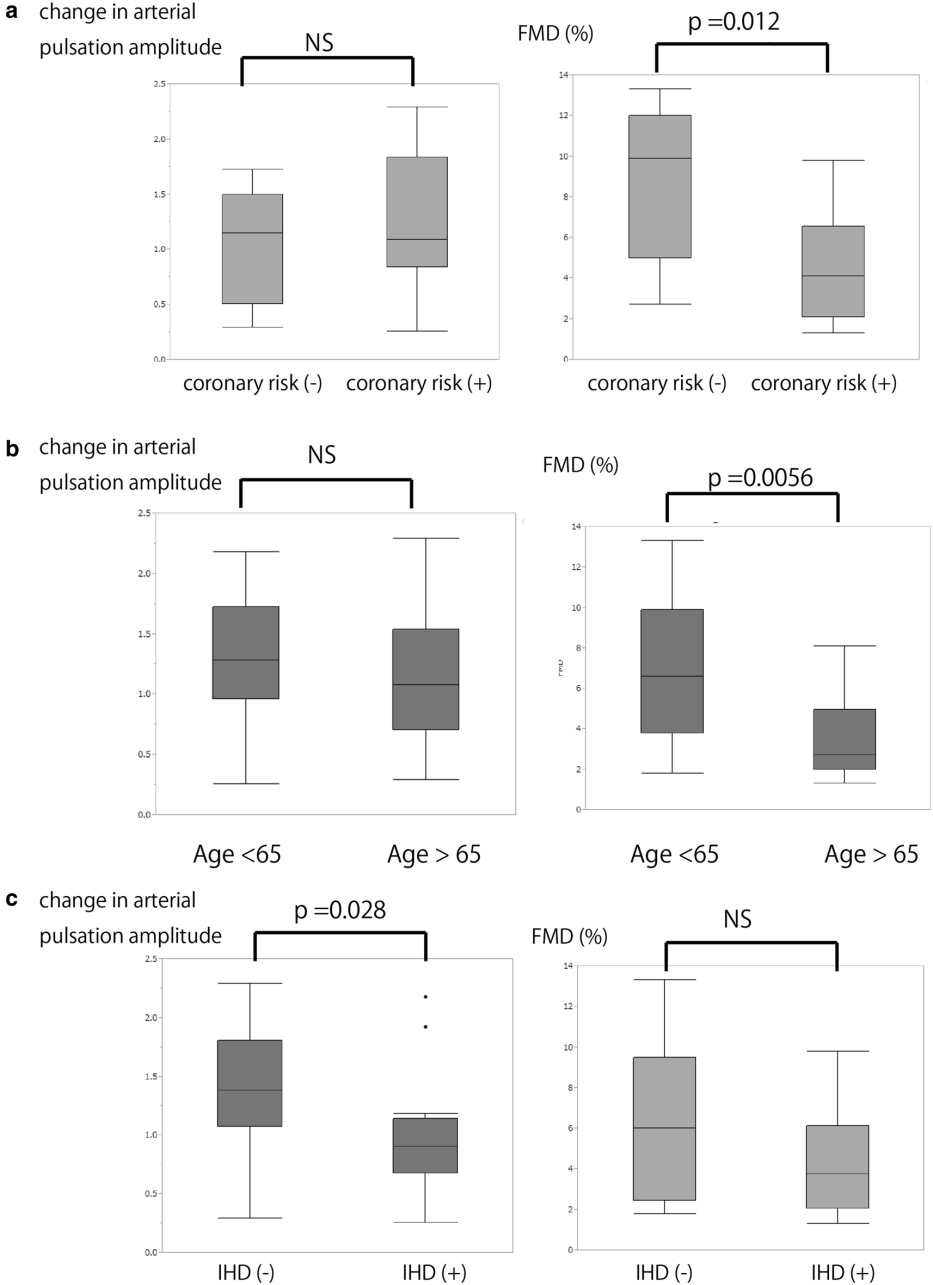
Comparisons of the change in arterial pulse amplitude and the value of flow-mediated dilation (FMD) between subjects with and without each coronary risks (**a**) or high age (**b**) or IHD (**c**)

We compared the change in arterial pulse amplitude and FMD between subjects with and without each of the coronary risk factors (diabetes, hypertension, hyperlipidemia) or high age (more than 65 years old) or IHD. There were significant differences in FMD with and without each of the coronary risk factors and the presence of high age, whereas there were no differences in the change in the arterial pulse amplitude (Fig. 3). By contrast, there was a marked difference in the change in arterial pulse amplitude between subjects with and without IHD (0.98 ± 0.53 and 1.37 ± 0.53, respectively; *p* = 0.028), whereas the value of FMD was not significantly different. In addition, the prevalence of IHD was significantly higher in the subjects with decreased arterial pulse amplitude during FMD than it was in those with increased arterial pulse amplitude (75 vs. 26.3 %). After adjusting for age, FMD, the value of arterial pulsation, coronary risk factors, and sex, the decrease in arterial pulse amplitude during FMD had significant predictive power for the presence of IHD (*p* = 0.0001).

## Discussion

In the present study, we observed an enhanced arterial pulse amplitude during FMD that was reduced in the presence of IHD. The enhancement of the arterial pulse amplitude was not considered to be due to the change in blood pressure (Naka et al. 2003), which has been reported to be a factor regulating arterial pulsation (Giannattasio and Mancia 2002), because the blood pressure was somewhat stable during FMD (Naka et al. 2006). The finding that the arterial pulse amplitude returned to the baseline level suggests that the arterial pulsation enhancement during FMD is only a temporary response induced by increased shear stress after cuff release. Karner et al. (1999) demonstrated a reduced shear stress in a distensible vessel model as compared with that in the rigid model. The enhancement of arterial pulse amplitude is presumed to reduce the load of shear stress, and it is therefore considered to be an adaptive response, whereas the opposite response, such as decreasing arterial pulse amplitude, corresponds to a maladaptive response.

Previously, Kamran et al. (2010) demonstrated that the PWV, a useful marker of arterial stiffness, changes during FMD, and that this change is correlated with the value of the FMD. These results suggest that some endothelial-derived factor produced during FMD, such as nitric oxide, affects vascular stiffness, resulting in a change in the behavior of the PWV. In a similar manner, the enhancement of the arterial pulse amplitude observed in the current study was supposed to be derived from the effect induced by increased shear stress during FMD. However, we could not detect any correlation between FMD and the change in arterial pulse amplitude, suggesting that the main contributor to the enhanced arterial pulse amplitude is not nitric oxide derived from stimulated endothelial cells and, therefore, there is another determining factor. Surprisingly, several cases in the present study demonstrated a decrease in arterial pulsation instead of an increase. This suggests that there may be a factor other than a dilating factor like nitric oxide that has significant potency to suppress arterial pulsation in the dysfunctional vessels. Indeed, shear stress increases stimulate the endothelial release of several vasoactive substances, such as prostaglandins, endothelial-derived hyperpolarizing factor, endothelin, and acetylcholine (Koller et al. 1993; Kuchan and Frangos 1993; Martin et al. 1996) and these factors had been reported to affect arterial stiffness (McEniery et al. 2003). On the other hand, Ramsey et al. (1995) observed the increase in brachial artery distensibility during FMD in similar manner and, it was lost in subjects with heart failure. The authors concluded that the absence of arterial pulsation enhancement during FMD in subjects with heart failure was derived from the impairment of blood flow. However, the decrease of arterial pulse amplitude observed in the current study cannot be explained by that mechanism.

Interestingly, there were marked differences in FMD between subjects with and without each coronary factor and high age, whereas there were no differences in the change in arterial pulse amplitude. These observations suggest that the change in arterial pulse amplitude is independent of these risk factors, by which the value of FMD is significantly affected. Therefore, the change in arterial pulse amplitude will present a different susceptibility to the progression of atherosclerosis that is independent of coronary risk factors. Thus, the change in arterial pulse amplitude may represent a novel coronary risk marker.

To investigate the determinant factor for the change in arterial pulse amplitude, we demonstrated the clear correlation between BMI and the change in arterial pulsation amplitude. It is somewhat paradoxical because many previous reports demonstrated the attenuation of vascular function in the presence of obesity (Bagi et al. 2012). However Higashi et al. (2003) reported similar results that lower BMI attenuated the response of endothelium-derived vasodilation and it was derived from the increase of oxidative stress in subjects with low BMI. Therefore, there may be some associations between obesity related factor and the response of arterial pulsation amplitude during FMD, however it warrants further investigations.

Alterations in wall shear stress have been implicated in the focal distribution of coronary artery disease (Dhawan et al. 2010). Atherosclerotic plaques predominantly form in regions of low shear stress, whereas regions of physiological moderate shear stress are generally protected in the early stage of atherosclerosis (Wentzel et al. 2012). On the other hand, advanced plaques that start to project into the lumen experience high shear stress at their most stenotic regions, which promotes plaque destabilization (Fukumoto et al. 2008). The vascular response to the altered shear stress is also a determinant of plaque stability and the progression of atherosclerosis (Koskinas et al. 2010). Expanded arterial remodeling occurs in exposed lesions with high shear stress, leading to physiological and adaptive arterial enlargement to preserve the lumen and, subsequently, to restore shear stress to a more physiological level (Dhawan et al. 2010). In the current study, we focused the vascular maladaptive response to increased shear stress as a coronary risk marker because the dysfunctional response finely corresponded to the presence of IHD. By contrast, Gori et al. (2008) demonstrated that the vascular maladaptive response to low shear stress also corresponds to the existence of IHD. However investigation is required to elucidate the more concise relationship between the vascular response to altered shear stress and the risk of atherosclerosis progression.

In the current study, we observed brachial arterial behavior. Endothelial function investigated at the brachial artery has been reported to be correlated with that in the coronary artery (Teragawa et al. 2005). Therefore, a dysfunctional brachial artery is thought to correspond to a decreased compensative potency of the coronary artery, which is related to coronary events.

In conclusion, enhancement of the arterial pulse amplitude during FMD is significantly suppressed by the presence of IHD, and the response is independent of the FMD. Further prospective studies to investigate the characteristics of this measurement modality in more detail are warranted.

### Limitations

The primary limitations of this study are the small sample size and the retrospective nature of the data collection. Therefore, the results should be confirmed in larger studies in more robust way. The administration of hemodynamically-effective medications was not well coordinated among the subjects and shear stress was not adjusted between each subject. In addition, there were several points (in particular, gender ratio and the ratio of hypertension) that were significantly different between subjects with and without IHD. However, gender difference and the presence of hypertension did not affect the value of the change in arterial pulse amplitude in the current study (Additional file 1: Figures S1, S2). The association between vascular injury and the change in the arterial pulse amplitude was demonstrated clearly and robustly. Furthermore, in the FMD protocol performed in the current study, the increase of shear stress is set appropriately for the evaluation of a nitric-oxide-derived response (Mullen et al. 2001). Therefore, differences in shear stress induction may be more potent for the enhanced arterial pulse amplitude during changes in shear stress (Pyke and Tschakovsky 2005).

The present study remains a preliminary report. We cannot confirm the basic characteristics of this measurement modality, including the intraclass correlation ratio of the measurements. Prospective studies are warranted for further investigation.

## Additional file

10.1186/s40064-016-2794-0 The difference in the change in arterial pulse amplitude between male and female or with and without hypertension.
