# Supplementary material for: Enhancement of arterial pulsation during flow-mediated dilation is impaired in the presence of ischemic heart disease
Source: Springerplus. 2016 Jul 16;5(1):1103. doi: 10.1186/s40064-016-2794-0 (PMC4947464; doi:10.1186/s40064-016-2794-0)
Supplement: Supplementary file 1 — 10.1186/s40064-016-2794-0 The difference in the change in arterial pulse amplitude between male and female or with and without hypertension. [file 40064_2016_2794_MOESM1_ESM.pptx]

## Slide 1
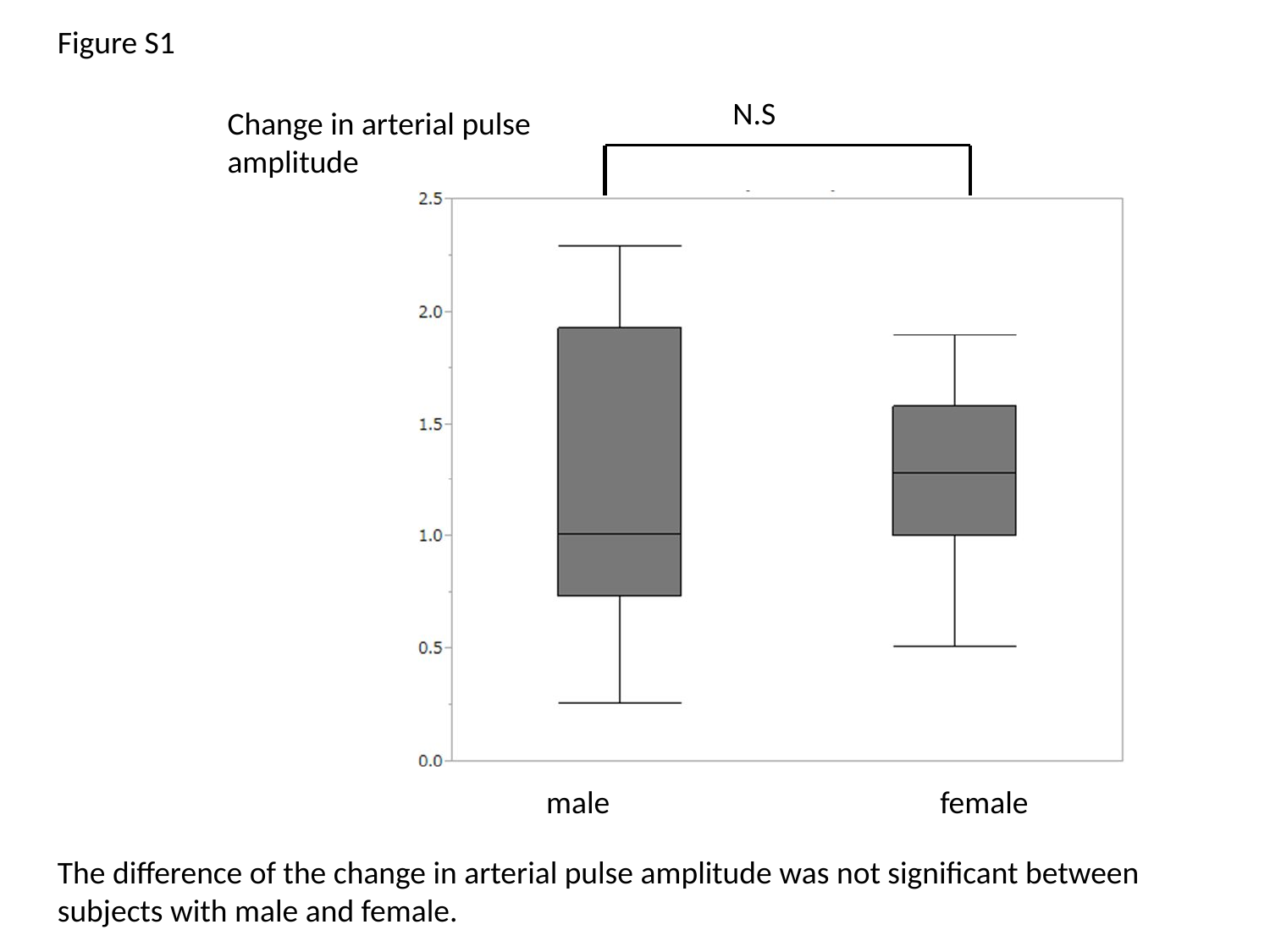

Figure S1
N.S
Change in arterial pulse amplitude
 male female
The difference of the change in arterial pulse amplitude was not significant between subjects with male and female.

## Slide 2
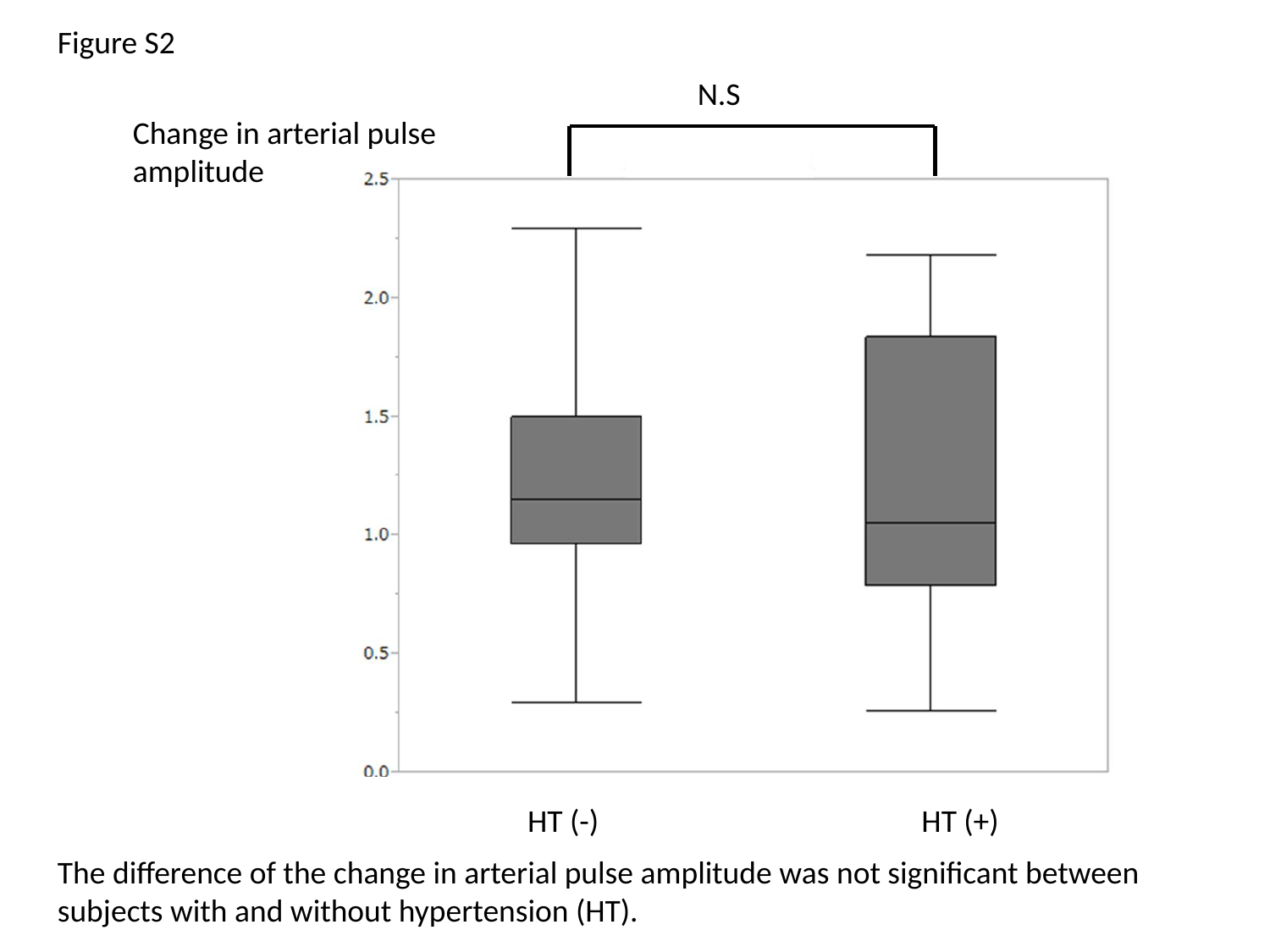

Figure S2
N.S
Change in arterial pulse amplitude
HT (-) HT (+)
The difference of the change in arterial pulse amplitude was not significant between subjects with and without hypertension (HT).
